# Magnesium Protects Cognitive Functions and Synaptic Plasticity in Streptozotocin-Induced Sporadic Alzheimer’s Model

**DOI:** 10.1371/journal.pone.0108645

**Published:** 2014-09-30

**Authors:** Zhi-Peng Xu, Li Li, Jian Bao, Zhi-Hao Wang, Juan Zeng, En-Jie Liu, Xiao-Guang Li, Rong-Xi Huang, Di Gao, Meng-Zhu Li, Yao Zhang, Gong-Ping Liu, Jian-Zhi Wang

**Affiliations:** 1 Department of Pathophysiology, Tongji Medical College, Huazhong University of Science and Technology, Wuhan, China; 2 Key Laboratory of Neurological Disease of National Education Ministry, Tongji Medical College, Huazhong University of Science and Technology, Wuhan, China; 3 Li Yuan Hospital, Tongji Medical College, Huazhong University of Science and Technology, Wuhan, China; Inserm U837, France

## Abstract

Alzheimer’s disease (AD) is characterized by profound synapse loss and impairments of learning and memory. Magnesium affects many biochemical mechanisms that are vital for neuronal properties and synaptic plasticity. Recent studies have demonstrated that the serum and brain magnesium levels are decreased in AD patients; however, the exact role of magnesium in AD pathogenesis remains unclear. Here, we found that the intraperitoneal administration of magnesium sulfate increased the brain magnesium levels and protected learning and memory capacities in streptozotocin-induced sporadic AD model rats. We also found that magnesium sulfate reversed impairments in long-term potentiation (LTP), dendritic abnormalities, and the impaired recruitment of synaptic proteins. Magnesium sulfate treatment also decreased tau hyperphosphorylation by increasing the inhibitory phosphorylation of GSK-3β at serine 9, thereby increasing the activity of Akt at Ser473 and PI3K at Tyr458/199, and improving insulin sensitivity. We conclude that magnesium treatment protects cognitive function and synaptic plasticity by inhibiting GSK-3β in sporadic AD model rats, which suggests a potential role for magnesium in AD therapy.

## Introduction

Alzheimer’s disease (AD), the most common form of dementia, is characterized by the progressive loss of neurons and synapses, the accumulation of intracellular neurofibrillary tangles that are primarily composed of hyperphosphorylated tau and extracellular senile plaques that are primarily composed of β-amyloid [1]–[3]. The molecular mechanisms underlying tau hyperphosphorylation and β-amyloid aggregation have been studied extensively [4], [5]; however, the exact etiopathogenesis of AD is poorly understood.

There following two forms of AD exist: familial (fAD) and sporadic (sAD). The great majority of AD cases occur sporadically at a late stage of life, while aging and metabolic disorders including Type 2 diabetes (T2DM) are the main non-genetic risk factors [6]. AD is associated with impaired glucose metabolism and insulin resistance in the brain. Impaired insulin signaling plays an important role in AD pathogenesis, and AD may be considered type-3 diabetes [7], [8]. Epidemiologic studies have also revealed that patients who suffer from T2DM have a two- to three-fold increased risk for AD [9]. Recently, it has been shown that diabetes increases the risk of dementia and the progression from mild cognitive impairment (MCI) to AD [10]. In addition, more than 80% of AD patients have T2DM or show abnormal blood glucose levels [11]. Diabetes causes the onset of amyloid pathology in a rabbit model and acts as a primary factor in inducing an early-stage AD phenotype [12]. T2DM and AD share several common abnormalities, including aging-related processes, high cholesterol levels, metabolic disorders, Aβ aggregation, tau protein phosphorylation, glycogen synthase kinase-3 (GSK-3) over-activation, insulin resistance and the induction of oxidative stress [12]–[15]. An intracerebroventricular (ICV) infusion of streptozotocin (STZ) is a valid experimental model to explore the etiology of sAD [16]; however, the mechanisms underlying ICV STZ-induced AD-like pathological changes remain elusive.

Magnesium plays an important role in a wide variety of critical cellular processes, including oxidative phosphorylation, glycolysis, cellular respiration and protein synthesis [17]. Magnesium depletion, particularly in the hippocampus, appears to represent an important pathogenic factor in AD [18]. A decreased magnesium level is found in various tissues of AD patients in clinical and laboratory studies [19]–[21]. A chronic reduction in dietary magnesium impairs memory [22], and the treatment of dementia patients with nutritional magnesium improves memory [23]. A causal relationship between low magnesium in hippocampal neurons and impairments in learning ability has been demonstrated in aged rats [24]. Recent studies have implicated that magnesium modulates the AβPP processing and that in the presence of high extracellular magnesium levels, AβPP processing stimulates the α-secretase cleavage pathway [25]. Moreover, treatment with a novel compound, magnesium-L-threonate (MgT), regulates NMDAR signaling, prevents synapse loss, and reverses memory deficits in aged rats [26] and AD model rats [27]. Interestingly, hypomagnesemia is a common feature in T2DM patients [28], and magnesium deficiency has been proposed as a risk factor for T2DM [29]. Therefore, magnesium is involved in AD and diabetes and may serve as a convergent point that links AD and diabetes.

The present study produced a sAD adult rat model using an ICV infusion of STZ and investigated the effects of the simultaneous supplementation of magnesium sulfate on ICV-STZ-induced AD-like pathological changes, memory deficits, and the underlying mechanisms of AD pathology. We found that the simultaneous intraperitoneal injection of magnesium sulfate restored brain magnesium levels, prevented ICV-STZ-induced memory impairments and reversed long-term potentiation (LTP) impairments with a concurrent increase in the expression of synapse-associated proteins and synaptic complexity. In addition, magnesium sulfate markedly decreased tau hyperphosphorylation at multiple AD sites in sAD rats by improving insulin sensitivity, and increasing the inhibitory phosphorylated GSK-3β (ser 9) through the activation of PI3K and Akt.

## Materials and Methods

### Animals and treatments

Three-month-old male Sprague-Dawley (SD) rats (weight 250±20 g) were obtained from the Experiment Animal Center of Tongji Medical College, Huazhong University of Science and Technology. All of the animal experiments were performed according to the “Policies on the Use of Animals and Humans in Neuroscience Research” from the Society for Neuroscience in 1995, and the Tongji Medical College Animal Experimental Ethics Committee approved all animal experiments. The animals were fed in a room under standard housing conditions (room temperature 24–27°C, humidity 60–65% and 12-h light-dark cycle) with free access to food and water.

The rats were anesthetized with 6% chloral hydrate (6 ml/kg, i.p.) and placed in a stereotaxic instrument (SR-6N; Narishige Scientific Instrument Laboratory, Tokyo, Japan). STZ (Sigma, St. Louis, MO, USA), dissolved in artificial cerebrospinal fluid (aCSF), was infused slowly bilaterally into the cerebroventricles of rats (10 µl on each site, final concentration of 3 mg/kg body weight) with the following coordinates: 0.8 mm anterior to posterior (AP) bregma, 1.5 mm midline to lateral (ML), and 4.0 mm dorsal to the ventral (DV) dura. The same volume of aCSF was infused as the vehicle control. Magnesium sulfate dissolved in normal saline was administered for seven consecutive days via intraperitoneal injection, and the vehicle control rats were injected with normal saline. The rats were divided randomly into the following six groups (n = 12 each): sham-operated control (Con), magnesium control (100 mg/kg Mg, i.p.), STZ group (3 mg/kg STZ, ICV), and STZ (3 mg/kg STZ, ICV) plus Mg groups (50 mg/kg, 100 mg/kg and 200 mg/kg, i.p.).

### Morris water maze

The water maze test was performed as previously described [30]. The water maze was conducted in a large circular black pool (160 cm in diameter) containing water (temperature at 24±2°C) that had been colored with a nontoxic black dye to contrast the rat. A 12-cm-diameter black-colored round platform was placed 1.5 cm below the water surface. All of the rats were placed in the water maze room 1 h before the water maze trial daily. The rats were given a maximum time of 60 s to find the hidden platform, and they were allowed to remain on the platform for 30 s. The rats were guided to land on the platform if they failed to find the platform within 60 s. The rats were given a daily session of four trials per day for six consecutive days. The swimming pathway and latency in locating the hidden platform were recorded for each trial. On the seventh day, the number of crossings and the percent time spent in the target quadrant were tested with the platform removed.

### Long-term potentiation

The rats were decapitated, and the brain was rapidly removed and placed in ice-cold aCSF continuously bubbled with 95% O_2_ and 5% CO_2_. The brain was cut using a vibratome (Leica, Wetzlar, Germany), and the obtained sections in the region of the hippocampus were incubated in the oxygenated aCSF at 30°C for 60 min and room temperature (20–25°C) until use. A slice was transferred to a submerged recording chamber and continuously perfused with aCSF (bubbled with 95% O_2_–5% CO_2_) at a rate of 1.5 ml/min. The temperature of the aCSF was maintained at 30–32°C using an in-line solution heater and temperature controller.

A multi-electrophysiological recording setup (MED64 system, Alpha Med Sciences, Japan) was used to record field excitatory postsynaptic potentials (fEPSP). The fEPSPs evoked at CA3-CA1 synapses were recorded from the dendritic layer of CA1 neurons by choosing an electrode in the Schaffer collateral pathway as the stimulating electrode [31]. A series of different stimulations between 5 mV and 30 mV were used to elicit the maximum fEPSP, and the 30–40% maximum stimulus intensity was selected as the basic stimulation. After a stable baseline of 30 min, LTP was induced using a standard high-frequency stimulation (HFS) paradigm consisting of four trains of 50 stimuli at 100 Hz (1 s each) repeated every 20 s. LTP was analyzed using the recording of fEPSP for 2 h after the conditioning stimuli, and population spikes were compared with the baseline.

### Atomic absorption spectroscopy

Rat brains were removed and the cerebral cortex and hippocampus were dissected for analysis. The brain regions were stored at −70°C until analysis using atomic absorption spectroscopy (AAS). Briefly, the brain tissues were placed in an incubator at 37°C and allowed to achieve a constant dry weight over approximately 24 h. Thereafter, the dry tissues were weighed and digested in concentrated nitric acid in glass centrifuge tubes for 1 h at 60°C and diluted 1∶10 with distilled deionized water before analysis. All samples produced clear digests, and the total magnesium in each sample was measured by flame AAS (Varian, SpctrAA-240FS, USA) using established and fully verified methods [32].

### Golgi staining

The Golgi staining protocol was performed as previously described [33]. Briefly, the rats were anesthetized after the behavioral tests and perfused through the aorta with approximately 250 ml of normal saline containing 0.5% sodium nitrite, followed by 500 ml of 4% formaldehyde solution and 500 ml Golgi fixative (5% chloral hydrate, 4% formaldehyde, and 5% potassium dichromate) for 2 h in the dark. The brains were incubated in the same Golgi fixative for 3 days and transferred to a silver solution containing 1% silver nitrate for 3 days in the dark. Coronal brain sections of hippocampal tissue were cut into 30-µm sections using a vibrating microtome (Leica, VT1000S, Germany). The numbers of spines and mushrooms were counted in at least 40 neurons in the hippocampus using a microscope (Olympus BX60, Tokyo, Japan).

### Immunohistochemistry

The rats were anesthetized and immediately perfused with 250 ml normal saline, followed by 500 ml of a 4% paraformaldehyde solution for 2 h. The brains were dissected, post-fixed for another 24 h, and sliced coronally at 30 µm using a vibrating microtome (Leica, VT1000S, Germany). The brain slices were soaked in PBS-0.5%Triton-0.3%H_2_O_2_ to remove the endogenous hydrogen peroxidase and blocked with 3% bovine serum albumin for 30 minutes. The slices were incubated for 48 h at 4°C with primary antibody, followed by incubation with a biotinylated secondary antibody for 1 h at 37°C. The immunoreaction was detected using horseradish peroxidase-labeled antibodies for 1 h in a 37°C oven and colored using the diaminobenzidine tetrachloride system (Bei Jing, ZSGB, 9032). The images were observed under a microscope (Nikon, 90i, Tokyo, Japan).

### Western blotting

Hippocampus were rapidly removed from the brains and homogenized in a buffer containing 10 mM Tris-Cl (pH 7.6), 1 mM Na_3_VO_4_, 50 mM NaF, 1 mM benzamidine, 1 mM EDTA, and 1 mM phenylmethylsulfonylfluoride (PMSF). The homogenates were mixed with one-third of sample buffer (200 mM Tris-HCl, 8% sodium dodecyl sulfate and 40% glycerol), boiled for 10 min, and centrifuged at 12,000 g for 10 min. The protein concentrations of the supernatants were measured using the BCA method. The same amount of protein was separated using SDS-polyacrylamide gel electrophoresis (10%) and transferred to a nitrocellulose membrane. The membranes were blocked in 3% non-fat milk for 1 h and incubated with primary antibodies at 4°C overnight. The membrane was incubated with secondary antibody conjugated to IRDye (800CW) for 1 h and visualized using the Odyssey Infrared Imaging System (LI-Cor Biosciences, Lincoln, NE, USA). The antibodies employed in the present study were listed in Table S1.

### Real-Time Quantitative PCR

Total RNA was isolated using TRIzol according to the manufacturer’s instruction (Invitrogen, Carlsbad, CA, USA). Then total RNA was reversely transcribed to cDNA using reverse transcription reagents kit (Toyobo Co., Ltd. Osaka, Japan). Fifty nanograms of cDNA was used for real-time PCR. For insulin (INS), the following primers were used: 5′-CAGCACCTTTGTGGTTCTCA-3′ (forward primer) and 5′-CAGTGCCAAGGTCTGAAGGT-3′ (reverse primer). For insulin receptor (INSR), the following primers were used: 5′-GCTTCTGCCAAGACCTTCAC-3′ (forward primer) and 5′-TAGGACAGGGTCCCAGACAC-3′ (reverse primer). For β-actin, 5′-CCCATCTATGAGGGTTACGC-3′ (forward primer) and 5′-TTTAATGTCACG CACGATTTC-3′ (reverse primer) were used. The following PCR cycle was used: 95°C/30 s, 40 cycles of 95°C /5 s, 58°C /30 s, and 72°C /60 s, and with subsequent melting curve analysis. The amplification and analysis were performed using a StepOnePlus Real-Time PCR Detection System (Life Technologies, NY, USA). All samples were compared using the relative CT method.

### Statistical analysis

All data are presented descriptively as the means ± standard deviation (SD) and analyzed using SPSS 17.0. One-way ANOVA was used, followed by least significant difference post hoc tests. The statistically significant of differences between the means for single comparisons were determined using the t-test. *P*<0.05 was considered statistically significant.

## Results

### Intraperitoneal magnesium supplement rescues ICV-STZ-induced memory deficits via elevations in brain magnesium

**Figure 1:**
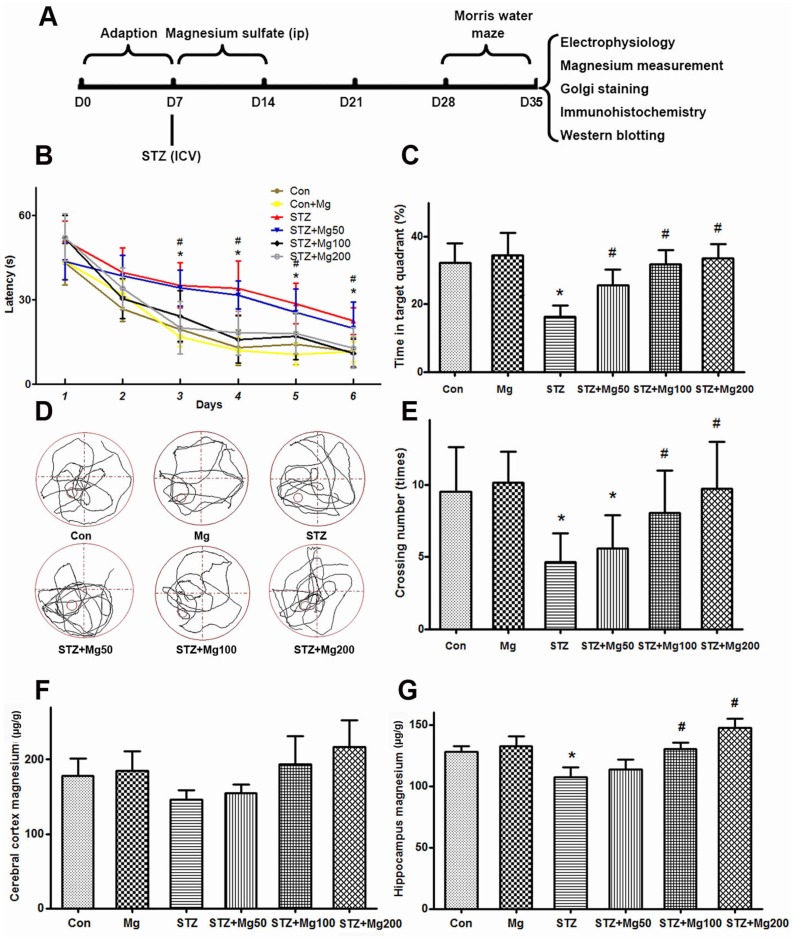
Intraperitoneal supplementation of magnesium rescues ICV-STZ-induced learning and memory deficits with elevation of brain magnesium level. The experiments were designed as shown in panel A. Rats were divided into six groups, i.e., sham-operated control (Con), sham-operated plus 100 mg/kg magnesium control (Mg), STZ ICV (STZ), STZ ICV plus 50 mg/kg magnesium (STZ+Mg50), STZ ICV plus 100 mg/kg magnesium (STZ+Mg100) and STZ ICV plus 200 mg/kg magnesium (STZ+Mg200) groups, as described in the Methods. During the 3^rd^ week after ICV-STZ treatment, the rats were trained in Morris watwe maze for six consecutive days to measure the learning capacity, and memory was tested on the 7^th^ day via removal of the hidden platform. The escape latencies to find the hidden platform were recorded daily (B). For the memory test, the time spent in the target quadrant (C), the swimming tracks (D) and numbers of crossings (E) in the target quadrant were calculated. The rats were sacrificed after the behavioral tests, and the magnesium levels in the cerebral cortex (F) and hippocampus (G) were measured. Data were presented as means ± SD. *P<0.05 versus the control group, *#P*<0.05 versus the STZ group.

A recent study showed that ICV-STZ (3 mg/kg) injections induced significant cognitive deficits during the 2^nd^ week and that these deficits persisted for up to the 14^th^ week in rats [34]. We used the Morris water maze to evaluate the learning and memory of rats during the 3^rd^ week after ICV-STZ treatment and investigated whether magnesium sulfate supplement could rescue the memory deficits. We found that the latency to find a hidden platform dramatically increased from the third day and that the crossing numbers and time spent in the platform quadrant significantly decreased at the seventh day in ICV-STZ-treated rats, which confirmed the STZ-induced memory deficits (Figs. 1A, B, D). A simultaneous supplement of magnesium sulfate (100 mg/kg and 200 mg/kg) efficiently attenuated the STZ-induced cognitive deficits (Fig. 1A–D). No significant difference was observed in the 50 mg/kg magnesium sulfate-treated group (Fig. 1A and B).

The brain and serum magnesium levels are significantly lower in AD patients than those in age-matched normal subjects [19], [20]. Furthermore, low magnesium is also observed in the brain of aged rats [24]. We observed that the magnesium levels decreased in the cerebral cortex and hippocampus in ICV-STZ-treated rats using the AAS method. Simultaneous magnesium sulfate (100 mg/kg and 200 mg/kg, but not 50 mg/kg) treatment significantly increased the brain magnesium levels in ICV-STZ-treated rats (Fig. 1E and F). Together with the above-mentioned behavioral results, we chose 100 mg/kg magnesium for the following experiments.

### Intraperitoneal magnesium supplement protects synapses from ICV-STZ-induced impairments

**Figure 2:**
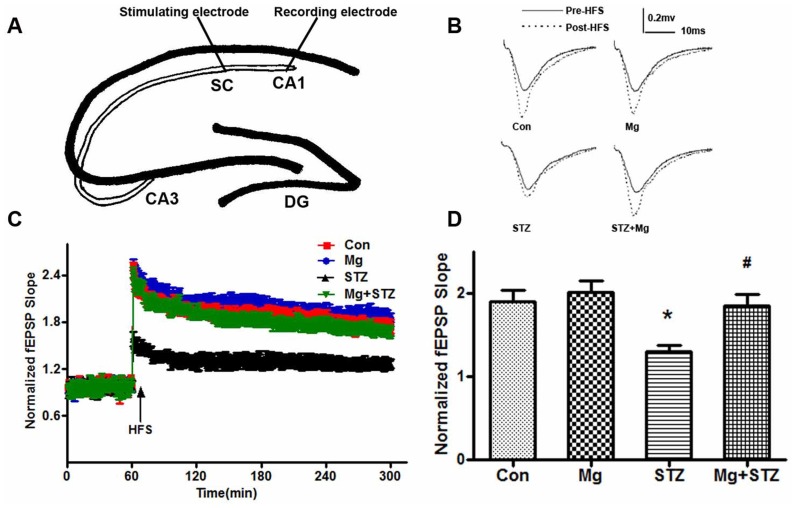
Magnesium reverses the LTP deficiency induced by ICV-STZ. Rats were divided into sham-operated control (Con), sham-operated plus 100 mg/kg magnesium control (Mg), and STZ ICV (STZ) or STZ ICV plus 100 mg/kg magnesium (STZ+Mg) groups. During the 3^rd^ week after ICV-STZ treatment, the hippocampal slices were prepared and an ideographic electrophysiology recording set-up with a stimulating electrode and recording electrode were placed in the CA3 and CA1 regions (A). The representative analog traces of evoked potentials before (solid line) and after (broken line) high-frequency stimulation (HFS) were recorded (B). Normalized field excitatory postsynaptic potential (fEPSP) slopes were measured in four groups (C), and the relative ratio of fEPSP increments after HFS (D) was calculated. Data were presented as means ± SD. *P<0.05 versus the control group, *#P*<0.05 versus the STZ group.

LTP contributes to synaptic plasticity and synaptic strength, which underlie learning and memory formation [35], [36]; therefore, we examined synaptic transmission using LTP recordings as previously described [31]. We found that the slope of excitatory post-synaptic potentials (EPSPs) increased approximately 1.3-fold following high-frequency stimulation (HFS) in the ICV-STZ-treated rats, which was much lower than the increase in vehicle-treated rats. In addition, simultaneous administration of magnesium sulfate supplements rescued the LTP deficit (Fig. 2A–D).

**Figure 3:**
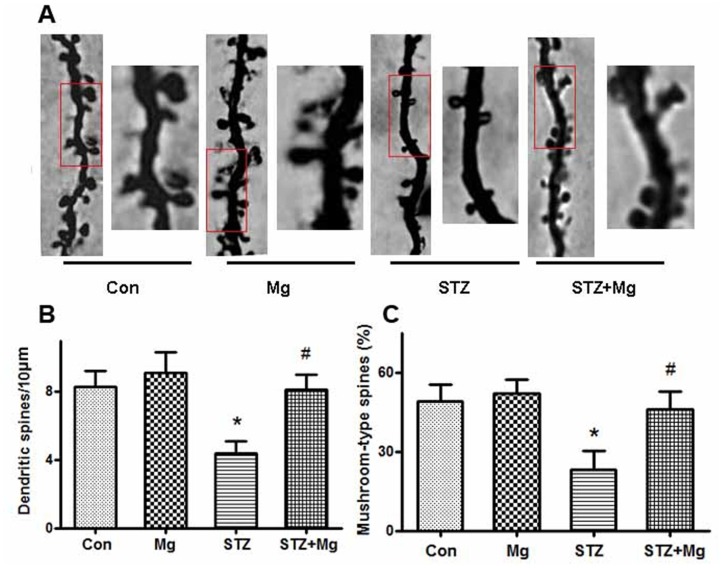
Magnesium reverses ICV-STZ-induced dendritic spines and synapse impairments. Rats were divided into sham-operated control (Con), sham-operated plus 100 mg/kg magnesium control (Mg), STZ ICV (STZ) or STZ ICV plus 100 mg/kg magnesium (STZ+Mg) groups and treated as shown in Figure 1A. The representative morphology alterations of neurons in the hippocampal CA1 regions were displayed by Golgi staining. Representative photomicrographs of primary dendrites in the hippocampal CA1 region were shown (A). Quantification of dendrite number (B) and mushroom-type dendrites (C) were calculated. The number of dendritic branches and mushroom percentage in the hippocampus of ICV-STZ-treated rats decreased markedly, and supplement of magnesium almost fully reversed the dendritic complexity. Data were presented as means ± SD. *P<0.05 versus the control group, *#P*<0.05 versus the STZ group.

Dendrite complexity and the morphologies of post-synaptic spines are critical components for learning and memory [37], [38]; therefore, we examined alterations in dendritic spines using Golgi staining. We found that the dendritic branches and mushroom-type spines in the hippocampus of ICV-STZ-treated rats decreased remarkably and that supplement treatment with magnesium sulfate almost fully reversed the number of dendritic branches and mushroom percentage (Fig. 3A–C). These results suggest that ICV-STZ damages postsynaptic plasticity and that magnesium sulfate preserves the morphological complexity of synapses.

**Figure 4:**
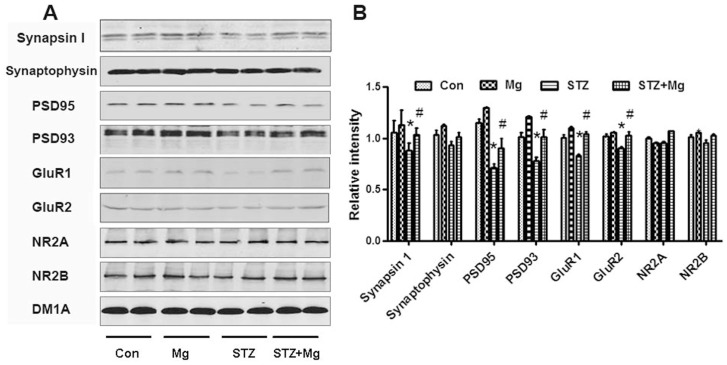
Magnesium increases synaptic proteins in the hippocampus of ICV-STZ-treated rats. Rats were divided into sham operated control (Con), sham operated plus 100 mg/kg magnesium control (Mg), STZ ICV (STZ) or STZ ICV plus 100 mg/kg magnesium (STZ+Mg) groups and treated as shown in Figure 1A. The levels of presynaptic and postsynaptic proteins in whole hippocampal extracts were measured using Western blotting (A) and quantitative analysis (B). The expression of synapsin I, PSD95, PSD93, GluR1 and GluR2 was significantly reduced in the hippocampus of ICV-STZ-treated rats, while supplementation of magnesium restored the levels. Levels of synaptophysin, NR2A and NR2B were not significantly changed. Data were presented as means ± SD. *P<0.05 versus the control group, *#P*<0.05 versus the STZ group.

Normal synaptic transmission is dependent on the stable expression of synaptic proteins. We detected several key synapse-associated proteins using Western blotting to further explore the molecular mechanisms underlying the protective effects of magnesium. The expression of presynaptic synapsin I was significantly reduced in the hippocampus of ICV-STZ-treated rats; however, the simultaneous supplementation of magnesium sulfate restored synapsin I levels, though the synaptophysin level remained unchanged (Fig. 4A and B). ICV-STZ treatment suppressed the expressions of postsynaptic PSD95, PSD93, GluR1 and GluR2, and the simultaneous addition of the magnesium sulfate supplement restored these levels (Fig. 4A and B). The NR2A and NR2B levels did not significantly change (Fig. 4A and B). These findings imply that a decrease in the expression of synapsin I, PSD95, PSD93, GluR1 and GluR2 contributes to the learning and memory deficits induced by ICV-STZ and that magnesium sulfate effectively prevents these impairments.

### Magnesium sulfate prevents ICV-STZ-induced tau hyperphosphorylation through the PI3K/Akt/GSK-3β pathway

**Figure 5:**
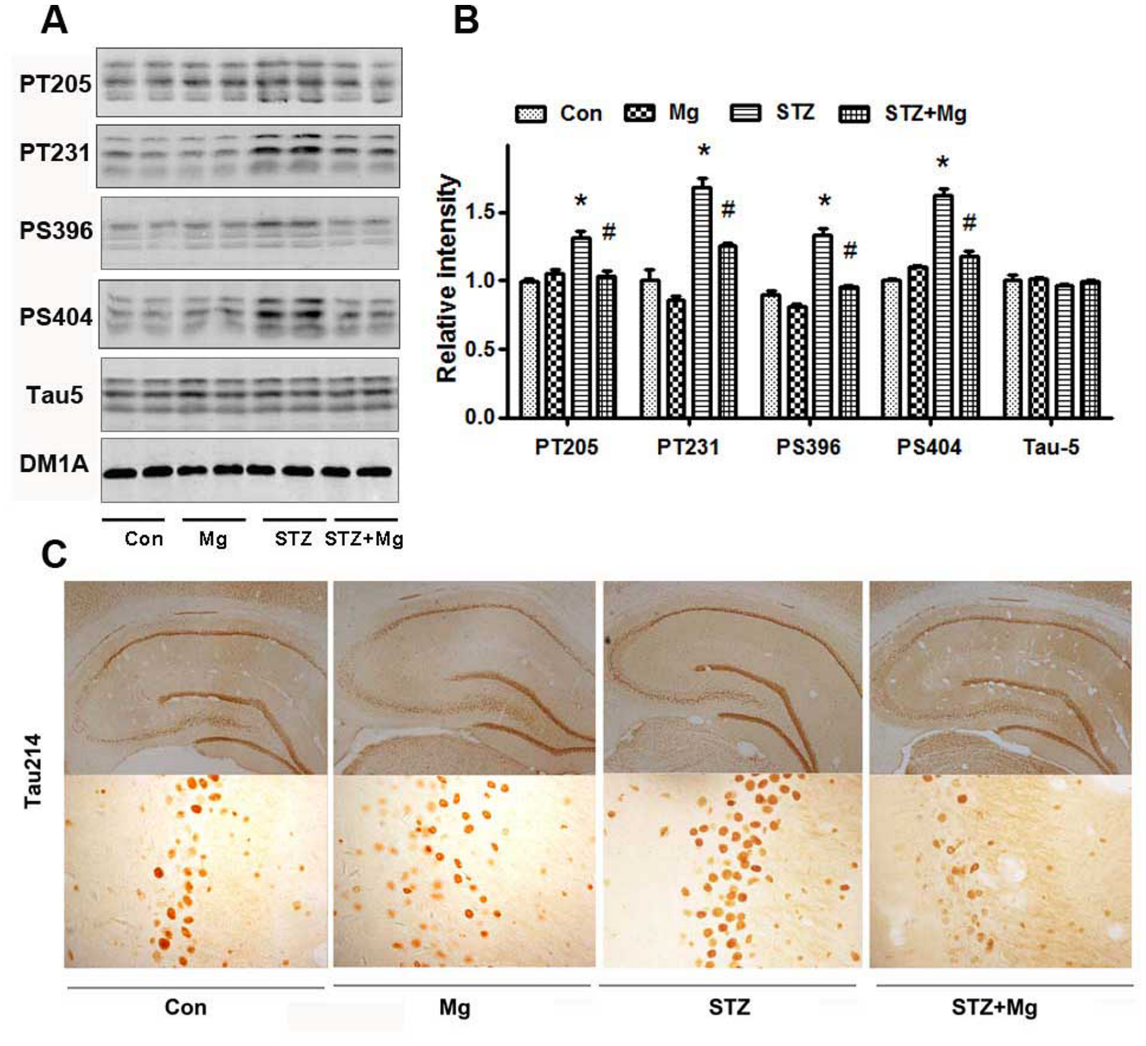
Magnesium prevents tau hyperphosphorylation in the hippocampus of ICV-STZ-treated rats. Rats were divided into sham-operated control (Con), sham-operated plus 100 mg/kg magnesium control (Mg), STZ ICV (STZ) or STZ ICV plus 100 mg/kg magnesium (STZ+Mg) groups and treated as shown in Figure 1A. The levels of total tau (tau-5) and phosphorylated tau at Thr205, Thr231, Ser396, and Ser404 in whole hippocampus extracts were measured using Western blotting (A) and quantitative analysis (B). The accumulation of Tau214 in the hippocampus neuron was detected using immunohistochemistry (C). The phosphorylation levels of tau at Thr205, Thr231, Ser396 and Ser404 sites increased significantly in the hippocampus of ICV-STZ-treated rats, while supplementation of magnesium attenuated tau hyperphosphorylation. The level of the total tau probed by tau-5 did not change. Attenuation of the STZ-induced tau hyperphosphorylation at Ser214 by magnesium was also detected using immunohistochemistry. Data were presented as means ± SD. *P<0.05 versus the control group, *#P*<0.05 versus the STZ group.

Tau hyperphosphorylation and accumulation play an important role in AD pathology [5]. In addition, ICV-STZ treatment in rats induces tau hyperphosphorylation [39]. We found that the tau phosphorylation levels at the Thr205, Thr231, Ser396 and Ser404 sites were significantly increased in the hippocampus of ICV-STZ-treated rats using a panel of site-specific antibodies (Fig. 5A and B). The simultaneous supplementation of magnesium sulfate attenuated tau hyperphosphorylation at these sites (Fig. 5A and B). The level of the total tau, as probed using tau-5, was not changed (Figs. 5A and B). The attenuation of the ICV-STZ-induced tau hyperphosphorylation at Ser214 by magnesium was also detected using immunohistochemistry (Fig. 5C).

**Figure 6:**
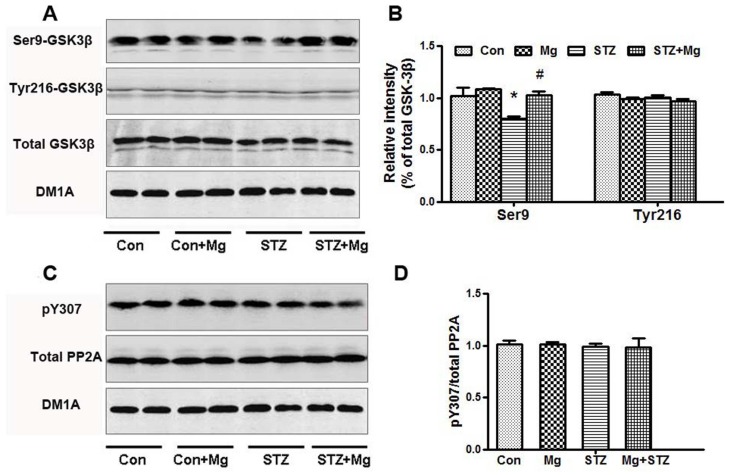
Magnesium suppresses GSK-3β with no effect on PP2A in the hippocampus of ICV-STZ-treated rats. Rats were divided into sham-operated control (Con), sham-operated plus 100 mg/kg magnesium control (Mg), STZ ICV (STZ) or STZ ICV plus 100 mg/kg magnesium (STZ+Mg) groups and treated as shown in Figure 1A. The total GSK-3β, GSK-3β (Ser9), GSK-3β (Tyr216), total PP2A and PP2A (pY307) levels in whole hippocampus extracts were measured using Western blotting (A, C) and quantitative analysis (B, D). The level of p-GSK-3β (Ser9) was significantly decreased in ICV-STZ-treated rats, and magnesium supplementation restored the levels. No alterations in total GSK-3β, p-GSK-3β (Y216), total PP2A and PP2A (pY307) levels were detected in these groups. Data were presented as means ± SD. *P<0.05 versus the control group, *#P*<0.05 versus the STZ group.

Protein kinases and phosphatases such as GSK-3β and PP2A regulate tau hyperphosphorylation [40]. We detected the activity of GSK-3β and PP2A using Western blotting to further explore the mechanisms underlying the ICV-STZ-induced impairments and magnesium protection. The total GSK-3β and p-GSK-3β (Y216) (the active form) levels were unchanged; however, the p-GSK-3β (Ser9) (the inactive form) level significantly decreased. Magnesium supplementation restored the p-GSK-3β (Ser9) level in ICV-STZ-treated rats (Fig. 6A and B). No alteration in PP2A activity was detected in these groups (Fig. 6C and D). These data suggest that GSK-3β inhibition contributes to the protective effect of magnesium sulfate in arresting ICV-STZ-induced tau hyperphosphorylation.

**Figure 7:**
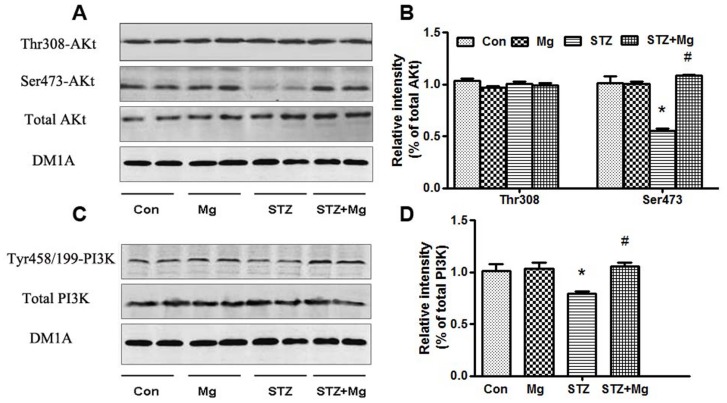
Magnesium stimulates the activity of Akt and PI3K in the hippocampus of ICV-STZ-treated rats. Rats were divided into sham-operated control (Con), sham-operated plus 100 mg/kg magnesium control (Mg), STZ ICV (STZ) or STZ ICV plus 100 mg/kg magnesium (STZ+Mg) groups and treated as shown in Figure 1A. The total Akt, Akt (Thr308), Akt (Ser473), total PI3K and PI3K (Tyr458/199) levels in whole hippocampus extracts were measured using Western blotting (A, C) and quantitative analysis (B, D), respectively. The p-Akt (Ser473) and p-PI3K (Tyr458/199) levels decreased significantly in ICV-STZ-treated rats, and these changes were fully restored by magnesium supplement. There was no change in total Akt, Akt (Thr308) and PI3K. Data were presented as means ± SD. *P<0.05 versus the control group, *#P*<0.05 versus the STZ group.

Phosphatidylinositide 3-kinase (PI3K)/Akt regulates the inhibitory phosphorylation of GSK-3β at Ser9 [41]. We measured the total amount and phosphorylation of Akt and PI3K, the upstream kinases regulator of GSK-3β, to investigate the role of PI3K/Akt in the neuroprotective effects of magnesium sulfate. Consistent with the finding of a previous study [42], the p-Akt (Ser473) and p-PI3K (Tyr458/199) levels decreased significantly in ICV-STZ-treated rats; however, no changes in total Akt, p-Akt (Thr308) and PI3K levels were observed (Fig. 7A–D). Magnesium sulfate supplementation almost fully restored these changes (Fig. 7A–D). These data suggest that the activation of the PI3k/Akt signaling pathway is involved in the neuroprotective effect of magnesium sulfate on ICV-STZ-treated rats.

**Figure 8:**
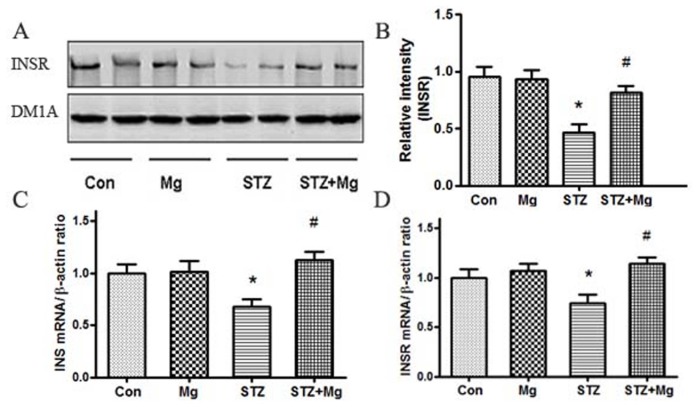
Magnesium reverses the INSR protein, INS and INSR mRNA levels in the hippocampus of ICV-STZ-treated rats. Rats were divided into sham-operated control (Con), sham-operated plus 100 mg/kg magnesium control (Mg), STZ ICV (STZ) or STZ ICV plus 100 mg/kg magnesium (STZ+Mg) groups, and treated as shown in Figure 1A. The protein level of INSR was measured using Western blotting (A, B) in whole hippocampus extracts. The mRNA levels of INS and INSR were measured using RT-PCR (C, D). The protein level of INSR, the mRNA levels of INS and INSR decreased significantly in ICV-STZ-treated rats, and these changes were reversed via magnesium supplement. Data were presented as means ± SD. *P<0.05 versus the control group, # P<0.05 versus the STZ group.

Brain insulin dysfunction plays a critical role in the pathogenesis of AD and leads to decreased PI3K/Akt signaling activity [43]. Therefore, we determined the protein level of INSR and the mRNA levels of INS and INSR. The protein level of INSR (Fig. 8A and B), the mRNA levels of INS and INSR (Fig. 8C and D) decreased significantly in ICV-STZ-treated rats, and these changes were reversed via magnesium sulfate supplementation.

## Discussion

AD is an age-related degenerative disease that is characterized by progressive dementia. AD therapies have been partially successful in the development of symptomatic treatments; however, these therapies have also encountered several failures in the development of disease-modifying cures [44], [45]. The progression of AD from the early stages of the neurodegenerative process to symptomatic stages occurs over a long period of time, but the disease rapidly causes devastating effects once cognitive impairments appear [46], [47]. These facts highlight the need to search for treatments that act on selective targets during the silent period of the disease, which are aimed at retarding disease progression toward dementia [48], [49].

Therefore, we investigated the effects of the simultaneous supplementation of magnesium sulfate on cognitive deficits in an ICV-STZ-induced rat model. The ICV administration of STZ is a well-established, validated, and widely accepted animal model of sAD, which develops many AD-like neuropathological features, including synaptic damage, amyloid-β deposition and tau hyperphosphorylation [50], [51]. Magnesium is required for many physiological processes, including insulin sensitivity, inflammatory responses, glucose metabolism, the regulation of cell proliferation and apoptosis, and defense against oxidative stress [52], [53]. Magnesium deficiency or imbalance has been implicated in AD pathogenesis [21], [22], [54], and an increase in brain magnesium improves learning and memory functions in aged rats [26]. Our study found that the simultaneous supplementation of magnesium sulfate effectively increased the brain magnesium levels and rescued ICV-STZ-induced learning and memory deficits.

Synaptic plasticity is a prerequisite of learning and memory and can be measured using alterations in LTP or synaptic morphology [55]. Increasing extracellular magnesium in the physiological range enhances synaptic plasticity in cultured hippocampal neurons, suggesting its role as positive regulator of synaptic plasticity [56]. Synaptic degeneration in AD was correlated with cognitive decline [57], [58]. We showed that the simultaneous supplementation of magnesium sulfate rescued LTP, preserved the morphological complexity of synapses and up-regulated the expression of synaptic proteins in ICV-STZ rats.

Tau hyperphosphorylation is the prelude of neurofibrillary tangle formation, which is positively correlated with the degree of clinical dementia. The ICV-STZ model shows neurodegenerative pathologies, including amyloid-β and hyperphosphorylated tau, which are similar to the brains of AD patients [59], [60]. Magnesium favors α-secretase cleavage pathways, which reduce amyloid-β [25], and we found that magnesium inhibited tau hyperphosphorylation in the sAD model.

The protein kinase and protein phosphatase (PP) GSK-3β and PP2A are the most implicated regulators of tau phosphorylation. As previously reported [61], [62], ICV-STZ induced the activation of GSK-3β. Tyr216 (the active form) and Ser9 (the inactive enzyme) phosphorylation regulate GSK-3β activity [63]. We identified that magnesium arrested STZ-induced GSK-3β activation via an increase in inhibitory phosphorylation at Ser9. A previous study also showed that magnesium, similar to zinc and lithium, is a potent inhibitor of GSK-3β [64]. GSK-3β is a downstream target of the PI3K/Akt signaling pathway [65], [66], and PI3K/Akt inactivation increases GSK-3β activity [42], [61], [62], [67]. Magnesium significantly enhances the activity of the PI3K/Akt pathway [68], [69]. Our data are consistent with recent studies [70], [71], which showed that ICV-STZ treatment decreases PI3K and Akt phosphorylation. We also found that the simultaneous supplementation of magnesium sulfate was prone to activate the PI3K/Akt pathway and inactivate GSK-3β.

ICV-STZ can induce an insulin resistant state in the brain and other similarities with human sAD [72]. In addition, deregulation of brain insulin and insulin receptor has been linked to the pathogenesis of AD [73]. In humans and in animal models, magnesium deficiency modulates insulin sensitivity, and may be associated with impaired insulin secretion [74]. The present results showed that magnesium could promote the protein expression of INSR, the mRNA levels of INS and INSR in ICV-STZ-induced rats. Our research indicates that brain insulin sensitivity could be mediated by magnesium, which might be related to PI3K/Akt signaling pathway in the sAD model.

In summary, we found that simultaneous intraperitoneal injections of magnesium sulfate significantly enhanced the brain magnesium levels, improved synaptic efficacy, and prevented memory and learning impairments through modifications of synaptic proteins and Tau phosphorylation in ICV-STZ rats. Our findings provide novel insights suggesting that magnesium treatment at the early stage may decrease the risk for cognitive impairment in AD.

## Supporting Information

Table S1 **Antibodies employed in the study.**
(DOC)
