# Supplementary material for: Magnesium Protects Cognitive Functions and Synaptic Plasticity in Streptozotocin-Induced Sporadic Alzheimer’s Model
Source: PLoS One. 2014 Sep 30;9(9):e108645. doi: 10.1371/journal.pone.0108645 (PMC4182554; doi:10.1371/journal.pone.0108645)
Supplement: Table S1 — Antibodies employed in the study. (DOC) [file pone.0108645.s001.doc]

**Table S1. Antibodies employed in the study**

| Antibody | Speciﬁcity | Dilution | Type | Company |
| --- | --- | --- | --- | --- |
| PSD93 | Total PSD93 | 1:1000 | Poly | Abcam |
| PSD95 | Total PSD95 | 1:1000 | Poly | Cell Signaling |
| GluR1 | Total GluR1 | 1:1000 | Poly | Millipore |
| GluR2 | Total GluR2 | 1:1000 | Mono | Millipore |
| NR2A | Total NR2A | 1:1000 | Poly | Abcam |
| NR2B | Total NR2B | 1:1000 | Poly | Abcam |
| Synapsin I | Total synapsin I | 1:1000 | Poly | Millipore |
| Synaptophysin | Total synaptophysin | 1:1000 | Mono | Sigma |
| PS396 | Phospho-tau at Ser396 | 1:1000 | Poly | Signalway Antibody |
| PS404 | Phospho-tau at Ser404 | 1:1000 | Poly | Signalway Antibody |
| PT205 | Phospho-tau at Thr205 | 1:1000 | Poly | Signalway Antibody |
| PT231 | Phospho-tau at Thr231 | 1:1000 | Poly | Signalway Antibody |
| Tau-5 | Total tau | 1:1000 | Mono | Millipore |
| GSK-3β | Total GSK-3β | 1:1000 | Mono | Signalway Antibody |
| p-GSK3β (Ser9) | Phospho-GSK-3β at Ser9 | 1:1000 | Poly | Cell Signaling |
| Tyr279/Tyr216-GSK3β | Phospho-GSK-3β at Tyr279/Tyr216 | 1:1000 | Poly | Upstate |
| PP2Ac | PP2A catalytic subunit (α and β isoforms) | 1:1000 | Mono | Millipore |
| p-PP2A (Y307) | p-PP2Ac at Tyr307 | 1:1000 | Poly | Abcam |
| Akt | Total AKt | 1:1000 | Poly | Cell Signaling |
| Ser473-Akt | Phospho-AKt at Ser473 | 1:1000 | Poly | Cell Signaling |
| Thr308-Akt | Phospho-AKt at Thr308 | 1:1000 | Poly | Abcam |
| PI3K | Total PI3K | 1:1000 | Poly | Cell Signaling |
| Tyr458/199-PI3K | PI3K at Tyr458/199 | 1:1000 | Poly | Cell Signaling |
| INSR | Insulin Receptor | 1:1000 | Poly | Abcam |
| DM1A | α-Tubulin | 1:1000 | Mono | Sigma |

Mono, monoclonal antibody; Poly, polyclonal antibody.
